# A simple and rapid simultaneous measurement strategy for optical rotatory dispersion and circular dichroism

**DOI:** 10.1038/s41377-024-01595-y

**Published:** 2024-09-11

**Authors:** Junjie Du

**Affiliations:** State Key Laboratory of Precision Spectroscopy, School of Physics and Electronic Science, East China Normal University, Shanghai, 200062 China

**Keywords:** Circular dichroism, Optoelectronic devices and components

## Abstract

A simple cavity-based technology capable of simultaneously measuring optical rotary dispersion and circular dichroism within milliseconds offers ultra-high sensitivity and unprecedented spectral resolution. This advancement holds significant potential for various biochemical applications, including drug development, clinical diagnosis, and food science and safety.

## Abstract (web summary)

A simple cavity-based technology capable of simultaneously measuring optical rotary dispersion and circular dichroism within milliseconds offers ultra-high sensitivity and unprecedented spectral resolution.

## Graphical abstract

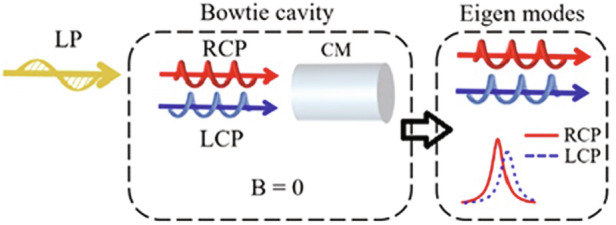

Chirality refers to the property of an object being non-superimposable on its mirror image. Our left and right hands exemplify this principle perfectly, thus distinguishing an object from its mirror image as being either left-handed or right-handed. Beyond the macroscopic examples, chirality is most strikingly manifested in the intricate structures of molecules and nanoscale systems. DNA, for instance, is a well-known chiral molecule with a right-handed twist, implicitly suggesting chirality’s potential link to the origins of life and its critical importance in drug development.

Identifying and characterizing chiral materials typically requires advanced analytical techniques that exploit physical property differences between right- and left-handed enantiomers. Chiroptical analysis is one of the most frequently used methods, utilizing the optical activity of chiral materials—specifically, the rotation of the polarization plane of linearly polarized light and the differential absorption of left and right circularly polarized light. The former is referred to as optical rotary dispersion (ORD), while the latter is known as circular dichroism (CD).

To overcome the limitations of conventional separate measurements in chiroptical analysis, research has focused on simultaneous measurement of ORD and CD using techniques such as interferometric Fourier-transform spectroscopy^1–4^ and dual-comb spectroscopy^5^. This is beneficial to enhancing accuracy and reliability in structural analyses through cross-validation, integrating the benefits of both methods while eliminating the need for sample-dependent method selection. Additionally, simultaneous measurement saves time and resources, optimizing overall efficiency when sequential measurement of ORD and CD has to be carried out.

A compelling question is whether optical microcavities can be utilized for the simultaneous measurement of absolute ORD and CD. This inquiry is motivated by the remarkable sensitivity demonstrated by cavity ring-down polarimetry in independently assessing ORD or CD^6–12^, attributed to the substantial enhancement of the effective light-matter interaction path length within a high-quality cavity. Ingeniously leveraging optical microcavities could therefore enable simultaneous, high-sensitivity measurements of ORD and CD.

Recently, Wenpeng Zhou et al. addressed this challenge in their latest work published in *elight*^13^, where they used a bowtie cavity with relatively low finesse to achieve ultra-high sensitivity of $\sim 2.7\times {10}^{-3}{\rm{deg}} /\sqrt{Hz}$ for ORD and $\sim 8.1\times {10}^{-6}/\sqrt{Hz}$ for CD. Notably, this approach did not compromise spectral resolution; instead, it achieved an unprecedented spectral resolution of 0.04 pm. The high spectral resolution is a critical indicator of spectrometer performance, crucial for obtaining finer fingerprint information of biomolecules. The temporal resolution of the spectrometer is also impressive, enabling millisecond-order fast measurements.

This work^13^ ingeniously combines optical microcavities with circular polarization beam splitters, forging a unique and innovative device. The microcavity serves two main purposes: it provides an environment for sufficient interaction between light and chiral molecules, and it creates significantly different electromagnetic responses due to the distinct cavity resonant frequencies between left and right circularly polarized light. When scanning the incident frequency, the resulting spectra for the left and right circularly polarized light exhibit sufficiently distinct differences, enabling the measurement of extremely weak ORD and CD. The researchers call this cavity-enhanced chiral eigenmode spectroscopy, as the transmitted light is separated into left and right circularly polarized components by a circular polarization beam splitter, with both components measured independently.

The approach presented in this article^13^ is simpler and more feasible for practical implementation. First, it uses only a single linearly polarized beam (the left panel in Fig. 1a), which makes this method robust against external perturbations such as mechanical vibration and thermal drift, and less dependent on precise alignment. In contrast, current cavity ring-down spectroscopy requires two beams and precise control of the angle between them (Fig. 1b). Second, the frequency does not need to be precisely locked to the cavity resonance frequency; instead, a more implementable frequency scanning strategy is employed. Finally, the resonance spectra corresponding to both circularly polarized components are measured separately, eliminating the need for an external magnetic field. Previous schemes for simultaneous measurement typically involve the use of an externally applied static magnetic field, which must be precisely modulated to create a large zero-signal bias for the beat frequency. The magnetic-free method excludes the magneto-optical effect and fully reflects the chirality of the materials.

**Fig. 1:**
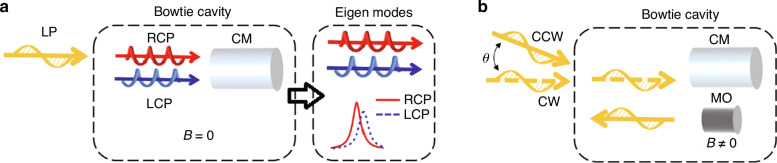
Comparison between cavity-enhanced chiral eigenmode spectroscopy method and cavity ring down polarimetry. **a** Cavity-enhanced chiral eigenmode spectroscopy method^13^. Only a single linearly polarized laser beam is input into the bowtie cavity with no external magnetic field. Instead of detecting the composite transmitted field, the left (dashed blue curve) and right (solid red curve) circularly polarized components are measured independently as a function of frequency. **b** Cavity ring-down polarimetry. Two linearly polarized laser beams making a precise angle of ${\rm{\theta }}$ are used to excite clockwise (CW) and counterclockwise (CCW) cavity resonant modes. Apart from chiral materials (CMs), the magneto-optical (MO) Faraday material and thus a magnetic field *B* are also used in bowtie cavities

With these combined advantages, this strategy^13^ may represent a new direction for developing higher-performance spectrometers. The cavity is a key component of the spectrometer and thus the focal point for future optimization. A suitable combination of finesse and quality factor could further enhance the spectrometer’s overall performance. The ultimate goal is to make this spectrometer a truly practical instrument, requiring comprehensive consideration of performance, cost, and operability. Greater efforts are worthwhile to be devoted to facilitating manufacturing, implementation, and ease of operation, enabling it to become a highly useful tool in biomolecular detection and pharmaceutical development.
